# Cancer-testis antigen lactate dehydrogenase C4 in hepatocellular carcinoma: a promising biomarker for early diagnosis, efficacy evaluation and prognosis prediction

**DOI:** 10.18632/aging.103879

**Published:** 2020-10-09

**Authors:** Zhaolei Cui, Yun Li, Yanni Gao, Lingying Kong, Yingfeng Lin, Yan Chen

**Affiliations:** 1 Laboratory of Biochemistry and Molecular Biology Research, Fujian Provincial Key Laboratory of Tumor Biotherapy, Department of Clinical Laboratory, Fujian Cancer Hospital and Fujian Medical University Cancer Hospital, Fuzhou 350014, Fujian, PR China; 2 Department of Blood Transfusion, The First Hospital of Fujian Medical University, Fuzhou 350009, Fujian, PR China; 3 Department of Pathology, Fujian University of Traditional Chinese Medicine Affiliated People’s Hospital, Fuzhou 350004, Fujian, PR China

**Keywords:** lactate dehydrogenase C, hepatocellular carcinoma, serum, exosome, prognosis

## Abstract

Expressions and clinical implications of cancer-testis antigen (CTA) lactate dehydrogenase (LDH)-C4 in hepatocellular carcinoma (HCC) have not been fully elucidated. Herein, expressions of *LDHC* mRNA in the serum and serum-derived exosomes of early-stage HCC patients were determined using qRT-PCR, and the expression of LDH-C4 protein in HCC tissues was detected using high-throughput tissue microarray analysis. It was found that positive rates of *LDHC* mRNA expressions in the serum and serum exosomes of HCC patients were 68% and 60%, respectively. The AUCs of serum and exosomal *LDHC* in differentiating HCC patients from healthy controls were 0.8382 and 0.9451, respectively. The serum and exosomal *LDHC* levels in HCC patients in the treatment group were higher than the levels in the preliminary diagnosis group, but lower than those in the recurrence group. Survival analysis showed that the expression of LDH-C4 was negatively correlated with the prognosis of HCC. The Cox regression analysis showed that an LDH-C4 level was an independent risk factor for the prognosis of HCC patients. Therefore, serum and exosomal *LDHC* can be used as a biomarker for early diagnosis, efficacy evaluation and recurrence prediction of HCC. Moreover, LDH-C4 can be used as an important reference indicator for monitoring the prognosis of HCC.

## INTRODUCTION

HCC is one of the most common malignant tumors of the digestive system [1]. According to the latest global cancer statistics, both the morbidity and mortality of liver cancer rank third among all tumors [2, 3]. However, most HCC patients, in fact, cannot be confirmed until tumors at various clinical stages metastasize to distinct organs, including intrahepatic and extrahepatic metastasis, posing recurrence and mortality risks to postoperative patients [4–6]. Therefore, improving the early diagnosis of HCC followed by timely targeted treatments is conducive to reducing the likelihood of metastasis in the patients. The first priority is the improvement of early detection biomarkers, so the survival of HCC patients can be extended with improved prognosis [7].

The CTA family is a group of antigens with strict tissue specificity, which is only expressed in the germinal epithelium of testis and some cancerous tissues in humans [8, 9]. It can be used as a putative biomarker for tumor diagnosis and the efficacy evaluation of immunotherapy [10–12]. LDH-C4, also known as LDHC or LDHX and encoded by *LDHC* mRNA, is the initially identified LDH isoenzyme only expressed in testis and tumors [13–15]. Human *LDHC* is located in chromosome 11p15.3-15.5, of which the full-length mRNA is 1179 bp with an ORF of 999 bp that encodes a 35 kDa C subunit. The polymerization of a homologous series of four identical C subunits can form the tetrameric protein, LDH-C4 zymoprotein, with catalytic activity [16]. LDH-C4 as a member of the CTA family features tissue-specific expressions only in the mature testis and sperm of healthy human males, which provides sufficient energy for sperm activities and is closely related to sperm development and motility physiologically [13–15].

Currently, few studies report biofunctions of LDH-C4 in tumor cells, and some find that *LDHC* mRNA is expressed in various tumors with positive rates of expressions of its spliceosomes up to 47% in lung cancer, 44% in melanoma, 35% in breast cancer and 15% in colon cancer [17]. Therefore, it is speculated that *LDHC* can be involved in the occurrence and development of these tumors. Other studies show that the positive rate of *LDHC* mRNA expression is 25% in non-small cell lung cancer (NSCLC) tissues, which is similar to the expression levels in adenocarcinoma and squamous cell carcinoma [18]. Wang et al. find that LDHC mRNA and its protein levels are up-regulated in renal cell carcinoma (RCC), and patients with positive LDHC expressions have a worse prognosis [19].

Exosome as a kind of extracellular vesicle (EV) with a diameter of 30-150 nm can be released by all living cells and also be detected in a tumor microenvironment [20, 21]. The latest evidences show that exosomes participate in the tumorigenesis by triggering angiogenesis and metastasis and suppressing anti-tumor immunity [22, 23]. Circulating exosomes in liquid biopsies can be used as non-invasive biomarkers for early detection, diagnosis and efficacy evaluation for cancer patients [24, 25]. In our previous work, IHC staining has been carried out to determine LDH-C4 expressions in 145 breast cancer tissue microarrays, and expression levels of peripheral serum and exosomal *LDHC* mRNAs in 75 breast cancer patients have been quantitated using RT-PCR analysis. The results preliminarily show that serum and exosomal *LDHC* mRNA can be utilized as an effective biomarker of breast cancer [26]. In this study, expressions of serum and exosomal *LDHC* mRNAs in stages I-II HCC were explored using qRT-PCR, and levels of LDH-C4 in HCC tissues were quantitated by high-throughput tissue microarray and IHC analyses, and correlations between LDH-C4 expression and clinical pathological characteristics and between the expression and the prognosis of HCC patients were analyzed.

## RESULTS

### Identification of isolated vesicles

**Figure 1:**
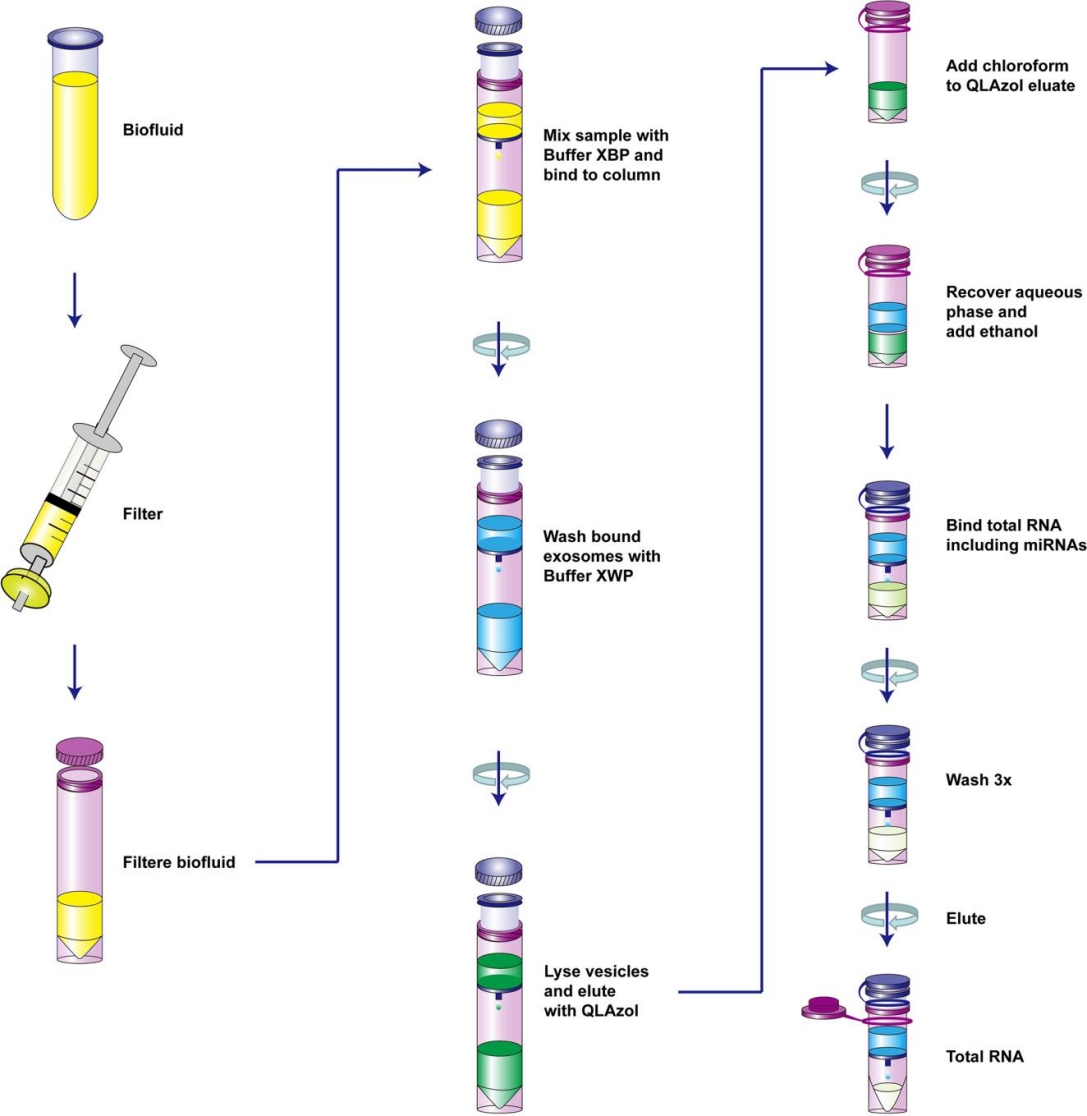
**The schematic diagram of the isolation of serum vesicles and RNA extraction.**

**Figure 2:**
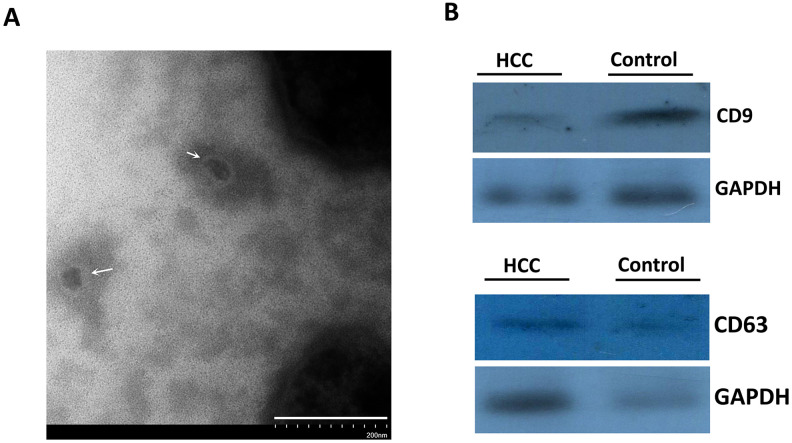
**The identification of isolated vesicles in the serum using TEM and immunoblotting.** (**A**) The morphology of serum exosomes under TEM. The white arrows indicated the isolated vesicles. (**B**) CD9 and CD63 proteins representing exosome markers were detected using western blot.

The extraction process was summarized in Figure 1. TEM showed that the size of exosomes derived from serum ranged from 30 to 150 nm in diameter and featured small vesicles with membrane structures, which were consistent with the morphological characteristics of exosomes (Figure 2A). To confirm whether exosomes were successfully isolated and purified, expressions of exosome marker proteins CD9 and CD63 were determined by western blot. The results showed that CD9 and CD63 could be detected in the eluate containing serum exosomes of both HCC patients and healthy controls (Figure 2B). This suggested that exosomes extraction was successful.

### Serum and exosomal *LDHC* acted as an early diagnostic biomarker for HCC

**Figure 3:**
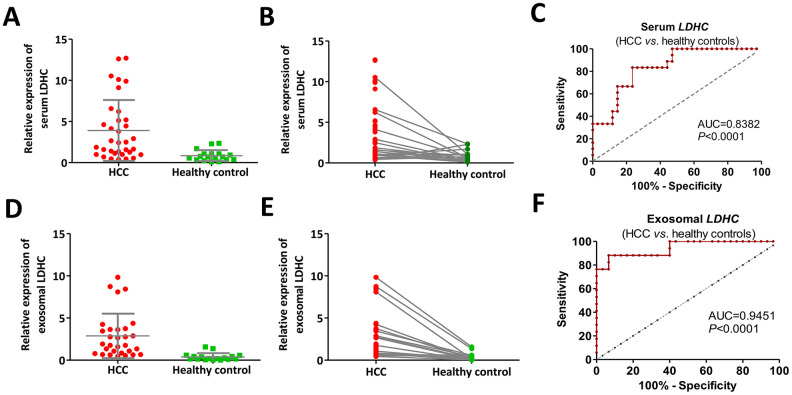
**Cancer-specific pattern of serum and exosomal *LDHC* expressions in early-stage HCC determined its diagnostic implication.** (**A**, **B**) Serum *LDHC* mRNA levels were significantly elevated in early-stage HCC serum samples compared with healthy individuals. (**C**) The ROC curve of serum *LDHC* mRNA for the identification of early-stage HCC. (**D**, **E**) Expressions of serum exosomal *LDHC* were elevated in early-stage HCC cases. (**F**) The ROC curve of serum exosomal *LDHC* in the diagnosis of early-stage HCC.

The qRT-PCR analysis showed that positive rates of *LDHC* mRNA expressions in serum and exosomes were 68% (34 / 50) and 60% (30 / 50) in early-stage HCC patients, and 18% (18 / 100) and 17% (17 / 100) in healthy controls, respectively. The results also showed that the average expression levels of serum and exosomal *LDHC* mRNAs in the preliminary diagnosis group were 4.64- and 14.28-fold higher than those in the healthy control group, respectively (Figure 3A, 3B, 3D, 3E). ROC curve analysis showed that the sensitivity, specificity and AUC of serum *LDHC* mRNA in distinguishing early-stage HCC patients from healthy controls were 83.3%, 76.5%, and 0.8382 (youden index = 0.598), respectively (Figure 3C); and the AUC of exosomal *LDHC* in distinguishing early-stage HCC patients from healthy controls reached 0.9451, under which the sensitivity and specificity were estimated to be 88.2% and 93.3%, respectively (youden index = 0.816) (Figure 3F). The above results suggested that serum and exosomal *LDHC* could be considered as a promising biomarker for early diagnosis of HCC.

### The correlations between the expression of *LDHC* in serum and exosomes and the clinicopathological characteristics of HCC patients

**Figure 4:**
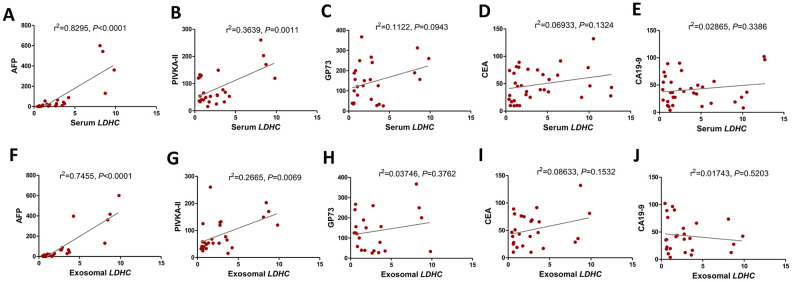
**Serum and exosomal *LDHC* expressions were correlated with clinicopathological characteristics in HCC.** Correlations between serum *LDHC* expressions and clinical indices encompassing (**A**) AFP, (**B**) PIVKA-II, (**C**) GP-73, (**D**) CEA, and (**E**) CA19-9 were analyzed. Serum exosomal *LDHC* expressions were associated with (**F**) AFP, and (**G**) PIVKA-II, but not correlated with the levels of (**H**) GP-73, (**I**) CEA, and (**J**) CA19-9 in patients with HCC.

The correlation analysis showed that serum *LDHC* levels were positively correlated with the levels of AFP (r^2^ = 0.8295, *P* < 0.0001) and PIVKA-II (r^2^ = 0.3639,*P* = 0.0011) (Figure 4A, 4B). Exosomal *LDHC* levels were correlated with the levels of AFP (r^2^ = 0.7455, *P* < 0.0001) and PIVKA-II (r^2^ = 0.2665, *P* < 0.0001) in HCC cases (Figure 4F, 4G). However, there were nonsignificant correlations between serum and exosomal *LDHC* levels and clinicopathological indicators such as gender, age, tumor size (all *P* > 0.05, data not shown), as well as serum levels of carcino-embryonic antigen (CEA), Golgi protein 73 (GP-73), and glycoprotein antigen 199 (CA19-9) (Figure 4C–4E, 4H–4J).

### Values of serum and exosomal *LDHC* in efficacy evaluation and recurrence monitoring

**Figure 5:**
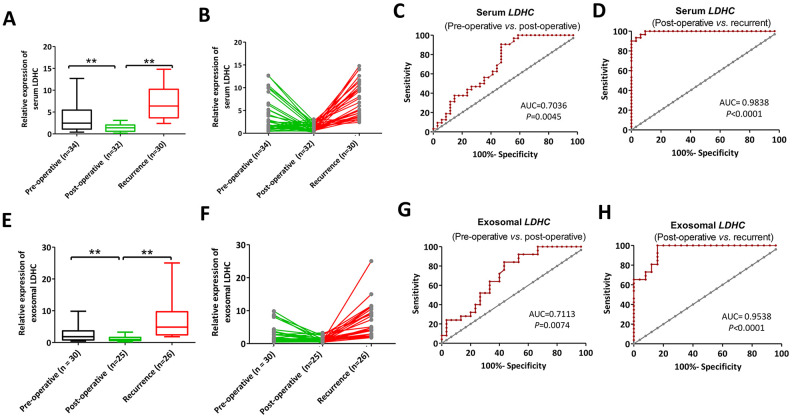
Serum (**A**, **B**) and exosomal (**C**, **D**) *LDHC* expressions in pre-operative, post-operative and recurrent patients with HCC. ROC curves of serum and exosomal *LDHC* mRNA expressions in the distinction between (**E**–**H**) pre-operative, post-operative, and recurrent cases. **P<0.01 *vs*. post-operative.

To explore the effects of *LDHC* mRNA on efficacy evaluation and recurrence monitoring, pre- and post-treatment *LDHC* mRNA expressions in the serum and exosomes of HCC patients were quantitated. It was found that the expression level of serum *LDHC* in the treatment group was significantly lower than that in the preliminary diagnosis group (AUC = 0.7036), while the average expression level of serum *LDHC* in the recurrence group was significantly higher than that in the treatment group (AUC = 0.9839) (Figure 5A–5D). Similarly, serum exosomal *LDHC* levels in the preliminary diagnosis group were significantly higher than the levels in the treatment group (AUC = 0.7113), and serum exosomal *LDHC* levels in the recurrence group were significantly higher than those in the treatment group (AUC = 0.9538) (Figure 5E–5H). These results suggested serum and exosomal *LDHC* could be used as an effective indicator for the evaluation of the efficacy of HCC-related treatments and recurrence prediction of patients.

### LDH-C4 as a prognostic marker of HCC

**Figure 6:**
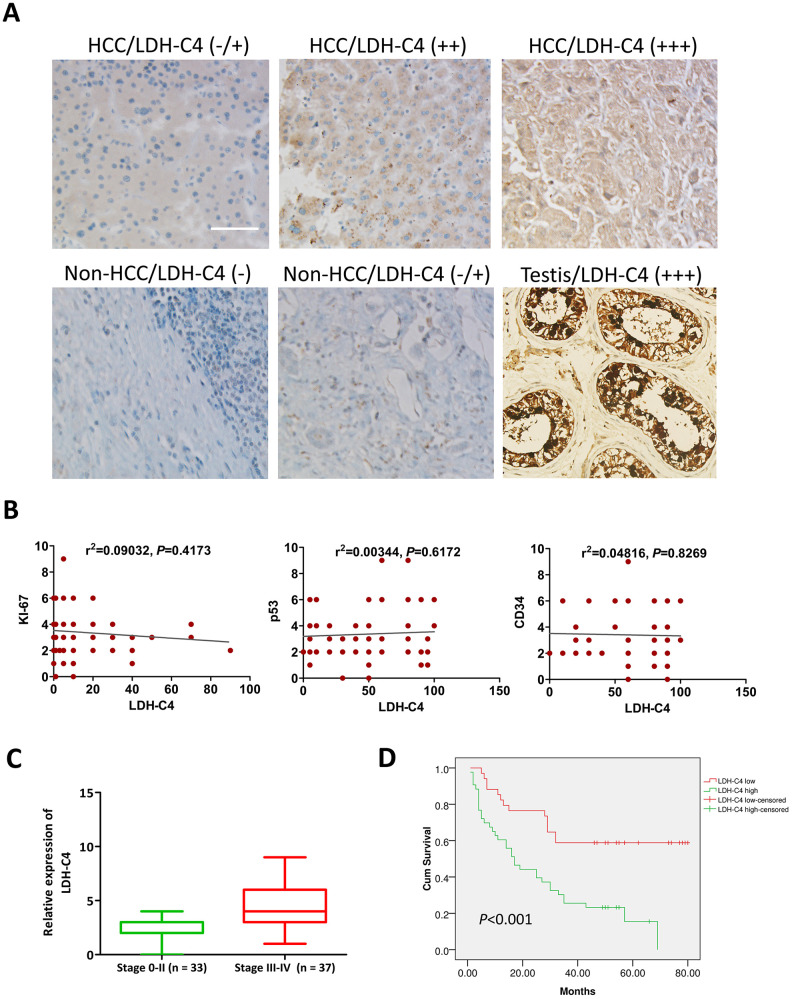
**LDH-C4 expressions in HCC tissues using IHC analysis.** (**A**) LDH-C4 levels were higher in HCC cancerous tissues than those in noncancerous tissues, and testis tissues were utilized as LDH-C4 positive controls (×200). (**B**) LDH-C4 levels were not significantly correlated with KI-67, p53 and CD34 expressions in HCC tissues. (**C**) LDH-C4 levels (rating scores) were higher in stage 0-II HCC patients than those in stage III-IV HCC patients. (**D**) The Kaplan-Meier analysis showed that LDH-C4 was negatively correlated with the prognosis of HCC patients, and higher levels of LDH-C4 implied worse prognosis.

It was found that LDH-C4 was mainly localized in the cytoplasm of HCC cells, which was significantly up-regulated in HCC tissues (Figure 6A). The proportion of LDH-C4 high expression (+ + / + + +) in HCC tissues accounted for 55.84% (43 / 77), which was significantly higher than that in para-carcinoma tissues (26.32%, 20/76) (*P* < 0.0001). The correlation analysis showed that LDH-C4 levels were positively correlated with clinical stage (*P* = 0.002) and mass size (*P* = 0.023) of HCC (Table 1), but not related to KI-67, p53 and CD34 levels (Figure 6B). Moreover, LDH-C4 staining score was higher in stage 0-II HCC patients than that in stage III-IV HCC patients (Figure 6C). The Kaplan-Meier analysis showed that the overall survival of patients in the LDH-C4 low expression group was significantly prolonged compared with the LDH-C4 high expression group, while the prognosis of patients in the LDH-C4 high expression group was worse than that in the low expression group (*P* < 0.001) (Figure 6D). The COX regression model analysis showed that LDH-C4 was an independent risk factor for HCC prognosis (*P* = 0.016) (Table 2).

**Table 1:** Correlations between LDH-C4 expressions and clinical characteristics in HCC patients.

**Clinicopathological features**	**Total case size**	**LDH-C4 expression (-/+)**	**LDH-C4 expression (++/+++)**	***P* value**
Age (years)				0.355
≤50	27	10	17	
>50	50	24	26	
Gender				0.468
Male	70	30	40	
Female	7	4	3	
Tumor size (cm)				0.023
≤5	32	19	13	
>5	45	15	30	
Clinical stage				0.002
Stage I+II	33	21	12	
Stage III+IV	37	10	27	

**Table 2:** The Cox regression analysis revealed independent risk factors for the prognosis of HCC patients.

**Risk factors**	**B value**	**SE**	**Wald**	***P* value**	**Exp(β)**	**Exp(β) 95%CI**	
						**Low**	**High**
Clinical stage	-1.159	0.721	2.584	0.108	0.314	0.076	1.289
T stage	-0.276	0.673	0.168	0.682	0.759	0.203	2.838
Tumor size	-0.198	0.426	0.216	0.642	0.820	0.356	1.890
LDH-C4 level	-0.926	0.383	5.857	0.016	0.396	0.187	0.839

### Expressions of LDHC in HCC tissues analyzed based on GEPIA data

**Figure 7:**
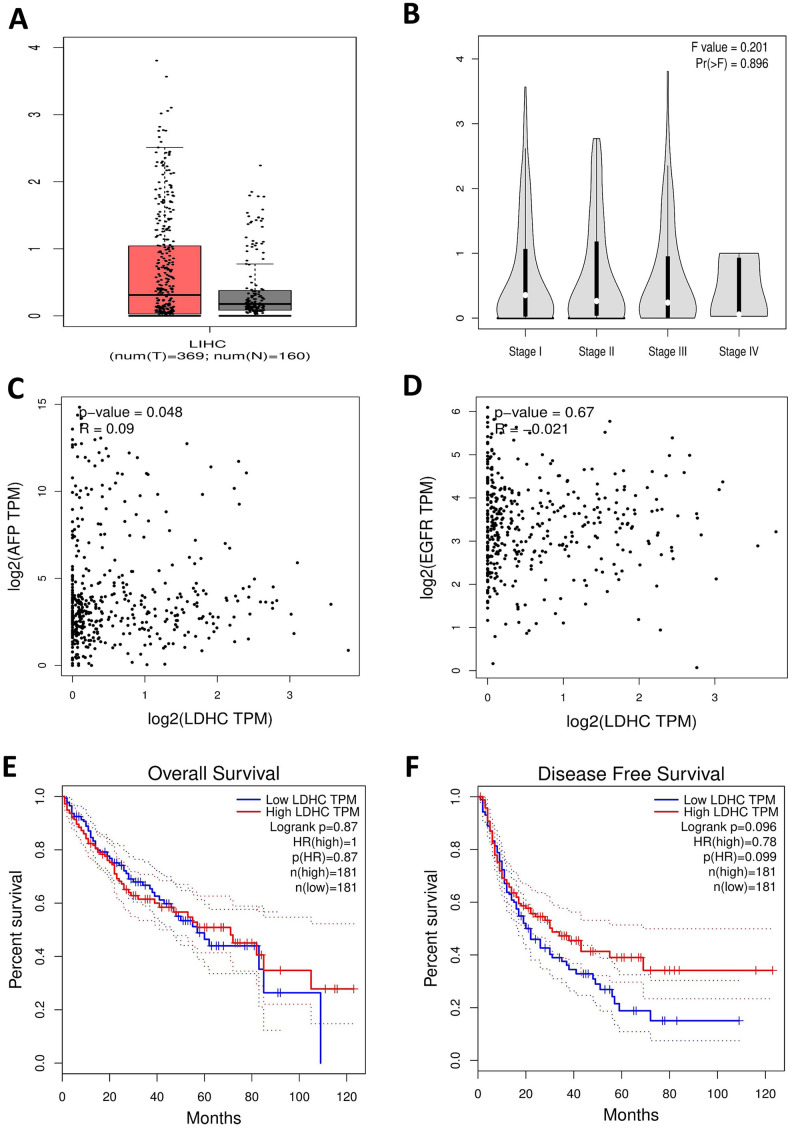
***LDHC* mRNA expressions in HCC tissues using TCGA data in the GEPIA database.** (**A**) The box plot of *LDHC* expression in HCC specimens and non-cancerous tissues. (**B**) *LDHC* expressions in cases of stages I-IV HCC. (**C**) *LDHC* expressions were positively correlated with AFP levels in HCC tissues. (**D**) *LDHC* levels were not correlated with EGFR expressions in HCC tissues. *LDHC* expressions were not correlated with the (**E**) OS and (**F**) DFS of HCC patients.

We also assessed the clinical significance of *LDHC* expressions in HCC based on GEPIA data that from TCGA. We found that *LDHC* levels were elevated in HCC tissues compared with non-cancer controls (Figure 7A), and also highly expressed in stage I-IV HCC patients (Figure 7B). The correlation analysis showed that *LDHC* expressions were positively associated with AFP levels (*P* = 0.048, Figure 7C), but not correlated with EGFR expressions in HCC patients (Figure 7D). The survival analysis showed that *LDHC* in HCC tissues was a nonsignificant predictor for OS and DFS of HCC patients (Figure 7E, 7F).

## DISCUSSION

Tumor cell growth is an energy-intensive process [27]. It has been proven that cancer cells including HCC cell lines exhibit disordered metabolism accompanied by releases of a substantial amount of energy through aerobic glycolysis even in the presence of adequate oxygen [28–31]. LDH-C4 is an enzyme specifically expressed in the testis and spermatozoa of birds and mammals, catalyzing the transformation of lactic acid to pyruvate via pathways of glucose metabolism [13–16]. As a member of the CTA family, LDH-C4 is also highly expressed in various malignant tumors such as lung cancer, renal cancer, and melanoma [18, 19, 32]. As LDH-C4 has a preference for lactate as a substrate [14], it is conceivable that LDH-C4 activation in cancers may rely on lactate for ATP production. This may partially explain the aberrant high expression level of LDHC in high ATP-consumption tumors. However, studies on roles of LDH-C4 in liver tumors have not been fully elucidated.

Exosomes are 30-150 nm diameter membrane vesicles released into the extracellular matrix upon fusion of intracellular multivesicular bodies with the cell membrane [20, 21]. It functions as a molecular warehouse carrying proteins, DNAs and RNAs and prevents their degradation during the cell cycle [33, 34]. Exosomes have been detected in the tumor microenvironment, and new evidence shows that their involvement in the angiogenesis, immune activity and metastasis of tumors results in the acceleration of tumor development [22, 23]. Therefore, circulating exosomes from liquid biopsies can be used as non-invasive biomarkers for early detection, diagnosis and efficacy monitoring of cancers [24, 25]. In this study, we for the first time explore serum and exosomal *LDHC* mRNA expressions and LDH-C4 protein expressions in HCC tissues using qRT-PCR, high-throughput tissue microarrays and IHC analyses, as well as their clinical implications in early diagnosis and prognosis prediction of the patients.

Previous studies confirm that an elevated concentration of free nucleic acids in the blood of tumor patients, compared with healthy people, can be explained by the delivery of nucleic acids by exosomes [33, 34]. This has been verified in our previous study which has showed that exosomes carry and release LDH-C4 molecules to the peripheral blood of breast cancer patients [26]. The present study for the first time elucidates that *LDHC* mRNA exists in the serum and exosomes of stages I-II HCC patients with a positive rate of 68% and 60% and the diagnostic sensitivity and specificity of higher than 80%. Our results have shown that serum and exosomal *LDHC* levels are positively associated with the recurrence of HCC and negatively correlated with treatments. All this indicates that serum and exosomal *LDHC* mRNA has high application values in early diagnosis, efficacy evaluation and recurrence monitoring of HCC. We have previously reported that the positive rates of LDH-C4, serum *LDHC* and exosomal *LDHC* are 91.55% (130 / 145), 91.66% (22 / 24) and 87.50% (21 / 24) in breast cancer tissues using a microarray analysis [26], which are consistent with the results of this study.

Currently, other members of the CTA family including MAGEC2 and KK-LC-1 have been reported to be prognostic indicators for HCC [35, 36]. To investigate the prognostic value of LDH-C4, we further analyze the relationship between the results of microarray analysis and clinical data of the patients, and find that LDH-C4 protein expressions in HCC tissues are correlated with clinical stage and tumor size of HCC. This suggests that LDH-C4 may participate in HCC progression. On top of that, the survival analysis shows that the OS of HCC patients who present high expression levels of LDH-C4 is shortened and their prognosis is poor, compared with the patients whose LDH-C4 levels are low. The Cox regression analysis has further confirmed that LDH-C4 is an independent risk factor for prognosis: patients with a high LDH-C4 level have a risk ratio of 2.5252 (1 / 0.396) times higher than the low LDH-C4 expression HCC cases. A latest research reports that expressions of *LDHC* mRNA and LDH-C4 protein are significantly up-regulated in RCC, and the prognosis of patients showing a positive LDH-C4 expression is worse [19], which is consistent with our current results and previous findings that LDH-C4 is a good indicator for prognosis monitoring of breast cancer patients [26]. However, the analysis based on GEPIA database (including the correlations between clinical stage and the survival) does not fully in line with our results of high-throughput tissue microarray analysis. The explanations can be as follows. Firstly, the survival analysis has been conducted based on LDH-C4 protein expressions determined by immunohistochemical analysis using rating scores. Secondly, due to protein post-translation modifications, *LDHC* mRNA expression levels using mRNA datasets in GEPIA can be inconsistent with its protein levels. On the other hand, *LDHC* expressions quantitated using the mRNA data in GEPIA database are positively associated with AFP levels of HCC patients (*P* = 0.048, *R* = 0.09), which is seemingly consistent with our findings in the serum and exosomes. However, as the R^2^ value in the correlation analysis is very small, the correlation between AFP and *LDHC* needs to be further verified.

To sum up, this study demonstrates serum and exosomal LDH-C4 expressions in cancerous tissues of HCC patients and its clinical implications. It can be deemed as a promising biomarker for early diagnosis, efficacy evaluation, recurrence monitoring and prognosis prediction of HCC. It is also expected to be a potential target for immunotherapy of HCC. However, the small sample size included in our study inevitably have somewhat biased the results. So our further study series are in need of more data from large-sample clinical trials for the verification of current findings.

## MATERIALS AND METHODS

### Clinical data

A total of 112 serum samples from HCC patients—including 50 cases of preliminarily diagnosed HCC (stages I-II), 32 treated cases and 30 recurrent ones—who were admitted to Fujian Cancer Hospital from December 2018 to December 2019 were collected. Another 100 serum samples from healthy volunteers were enrolled in the control group. All serum samples were collected with the approval of the ethics committee at our hospital (ethical grant No. SQ2015-049-01). Commercial high-throughput HCC microarray (HLivH180Su10, contained 93 HCC cases and 87 adjacent non-cancerous tissues) was provided by Shanghai Outdo Biotech Co., Ltd. (Shanghai, China), and only 77 samples from HCC cancer tissues and 76 from adjacent non-cancerous tissues were used. Among the 77 cancer patients consisting of 70 males and 7 females aged from 21 to 75 years with an average age of 45.32 ± 5.18 years, 33 cases were diagnosed as stage T1 + T2 (or stage I + II) HCC and 37 as stage T3 + T4 (or stage III + IV) HCC (unavailable data from 7 cases were not included). The tumor stage classification was guided by the TNM stage criteria of primary hepatocellular carcinoma (7^th^ edition) in the American Joint Committee on Cancer (AJCC) / the Union for International Cancer Control (UICC) staging in 2010. All cancer tissues were confirmed by pathological examination. The enrolled patients had complete clinical data and follow-up information. The operation time ranged from January 2007 to November 2009, and the follow-up period varied between 4 and 7 years from September 2013.

### Vesicle isolation and identification by western blot

Serum exosomes were extracted using an exoRNeasy Serum/Plasma Midi Kit (QIAGEN, Catalog No.77044) (Part I: vesicle isolation). The extracted exosomes were identified by transmission electron microscope (TEM). The detailed processes were described in our previous study [26]. Briefly, after total proteins extracted from the exosome eluate were lysed using RIPA lysis buffer, they were separated by gel electrophoresis (SDS-PAGE, 12% gels) at 80 V constant pressure and transferred to PVDF membranes at 250 mA constant current. TBST containing 5% skimmed milk powder was used for blocking the PVDF membranes overnight. Rabbit anti human CD9 monoclonal primary antibody (Abcam, Catalog No.ab92726; 1 : 300), rabbit anti human CD63 monoclonal primary antibody (Abcam, Catalog No.ab217345; 1 : 100), and mouse anti human GAPDH monoclonal primary antibody (Beyotime, Catalog No. AF0006; 1 : 1000) were added for incubation for 4 h at room temperature. Goat anti mouse / rabbit IgG (1 : 10000) with horseradish peroxidase (HRP) was added before a 2-h incubation at room temperature. ECL luminescent solution was employed for visualizing the protein bands which were subsequently photographed. Each experiment was repeated three times.

### Real-time qRT-PCR

Total RNAs were extracted from serum and serum-derived exosomes respectively using the MiRNeasy Kit (QIAGEN, Catalog No.217184) and exoRNeasy Serum/Plasma Midi Kit (Part II: RNA isolation, Figure 1), according to the manufacturer’s instruction. The serum and exosomal RNAs were reversely transcripted into cDNAs using the Transcriptor first strand cDNA synthesis Kit (Roche) according to the manufacturer’s instruction. The primer sequences of *LDHC* and *GAPDH* were previously described [26]: *LDHC*-F: 5'-TCATTCCTGCCATAGTCCA-3', *LDHC*-R: 5'-CAATTACACGAGTTACAGGTA-3'; *GAPDH-*F: 5'-TCGACAGTCAGCCGCATCTTCTTT-3'; and *GAPDH-*R: 5'-ACCAAATCCGTTGACTCCGACCTT-3'. Levels of *LDHC* mRNAs were determined using the ABI7500 fluorescence quantitative PCR detector and SYBR green real time PCR master mix (ROX) kit under the following conditions: denaturation at 95 °C for 10 min, then 95 °C for 15 s, 40 cycles, and finally 60 °C for 1 min. Subsequently, relative expressions of *LDHC* mRNAs were calculated using the 2^−ΔΔCt^ method normalized to *GAPDH*.

### Immunohistochemical analysis

The immunohistochemical analysis was performed using an EliVision^TM^ Plus Two-Step Test Kit. The process was described in our previous study [37]. Briefly, wax-embedded tissues were dewaxed in xylene and rehydrated in a series of ethanol solutions. They were washed and treated with H_2_O_2_ for 10 min. T The antigen was extracted by heating citric acid in a boiling-water bath at 100-120 °C and high pressure for 3 min. Sections were incubated in the diluted (1 : 50) rabbit anti human LDHC monoclonal antibody (Abcam, Cat.no.ab52747) at 37 °C for 90 min, washed with PBS, and incubated with goat anti-rabbit antibody for 30 min. The sections were stained with DAB for 2 min, and hematoxylin was used for contrast staining.

The proportion of LDH-C4-positive cells in each sample was scored as follows. A positive rate of less than 5% was scored as 0, the rate of more than 5% and less than 25% was scored as 1, the rate of more than 25% and less than 50% as 2, and that of more than 50% as 3. The staining severity was graded according to the final score for LDH-C4 expressions based on the positive rates: 0 point was rated as “−”, 1-2 points as “+”, 3-5 points as “+ +”, and 6-9 points as “+ + +”. Among them, “− / +” was defined as negative or low expression and “+ + ~ + + +” as high expression. All sections were judged by two experienced pathologists using a double-blind method.

### Bioinformatics analysis of available databases

*LDHC* mRNA expressions in HCC were also analyzed using available TCGA data in GEPIA (http://gepia.cancer-pku.cn/). A Log_2_FC cutoff value (Log_2_FC = 2, *P =* 0.01) was used to distinguish a high *LDHC* expression group from a low expression group. For the survival analysis, high and low *LDHC* expression groups were identified using a group cutoff of the median.

### Statistical analyses

Data were analyzed using SPSS 16.0 software. Measurement data were presented as mean ± standard deviation (SD). We studied the fulfillment of the normality and homogeneity of variance assumption prior to the application of Student's *t*-test for comparisons between groups. Kaplan-Meier survival curve was used to analyze the overall survival of HCC patients. The correlations between LDH-C4 expressions and clinicopathological features and between the expression and the prognosis of HCC patients were analyzed using chi-square test and Spearman’s rho test. A Cox proportional hazard regression model was established to analyze independent prognostic risk factors of HCC. A *P* value of less than 0.05 was considered statistically significant.
